# Green Nanomaterials for Smart Textiles Dedicated to Environmental and Biomedical Applications

**DOI:** 10.3390/ma16114075

**Published:** 2023-05-30

**Authors:** Melania Popescu, Camelia Ungureanu

**Affiliations:** 1 National Institute for Research and Development in Microtechnologies—IMT Bucharest, 126A Erou Iancu Nicolae Street, 077190 Bucharest, Romania; b.melania06@gmail.com; 2 General Chemistry Department, University “Politehnica” of Bucharest, Gheorghe Polizu Street, 1-7, 011061 Bucharest, Romania

**Keywords:** green nanomaterials, smart textiles, environmental applications, biomedical applications, nanoparticles

## Abstract

Smart textiles recently reaped significant attention owing to their potential applications in various fields, such as environmental and biomedical monitoring. Integrating green nanomaterials into smart textiles can enhance their functionality and sustainability. This review will outline recent advancements in smart textiles incorporating green nanomaterials for environmental and biomedical applications. The article highlights green nanomaterials’ synthesis, characterization, and applications in smart textile development. We discuss the challenges and limitations of using green nanomaterials in smart textiles and future perspectives for developing environmentally friendly and biocompatible smart textiles.

## 1. Introduction

Significant advances have been reported lately for smart textiles, alongside progress in materials science and nanotechnology [1]. Smart textiles, also named smart fabrics or e-textiles, are designed with integrated electronic components, sensors, and other technologies. These components can be used to monitor, transmit, and receive data, and can be embedded in textiles in various ways, such as by weaving or printing them directly onto the fabric [2]. Based on how they react to the environment, they can be classified into passive smart textiles (can sense the environment), active smart textiles (can sense and react to stimuli from the environment), and very smart textiles (can sense, react, and adapt their behaviour based on the received stimuli) [1,3]. Based on their functionality, smart textiles can also be classified into sensing, actuating, energy harvesting, and communicating [4]. Thus, smart textiles can be designed to detect and measure various physical and chemical parameters (i.e., temperature, pressure, humidity, and gas concentration), and can be employed in healthcare and environmental monitoring [5]. The actuating smart textiles can respond to external stimuli (i.e., heat or light) and change their properties or shape accordingly [6]. Smart textiles can also be designed to capture and store energy from external sources, such as sunlight or body heat, and use it to power other devices or sensors [7,8]. Finally, they can be designed to transmit and receive data wirelessly, allowing them to be integrated into more extensive networks or systems [9].

In the environmental sector, smart textiles can be used to monitor environmental conditions (i.e., temperature, humidity, and light). They can also be used in construction for indoor air quality monitoring and controlling heating and cooling systems [10].

In healthcare, smart textiles can be employed to monitor a range of health parameters (i.e., heart rate, respiration, and body temperature), and to deliver drugs or other therapeutic agents directly to the body. For example, smart textiles can be used as wearable sensors to monitor patients with chronic conditions, such as diabetes, and to alert healthcare providers to potential problems [11,12].

Smart textiles can represent a sustainable choice compared to conventional textiles due to their unique properties and functionality. For example, the fabrics can be designed to use fewer energy and water resources during production and use. They can be made more durable by integrating specific functions such as self-cleaning, thus reducing the need for frequent washing, which generates textile waste. Additionally, they can be combined with renewable energy sources such as solar cells, which can reduce reliance on non-renewable energy sources [8,13,14].

However, not all smart textiles are necessarily more sustainable than conventional textiles. Textile sustainability depends on the materials used, the manufacturing process, and the textile’s end-of-life options. Hence, the sustainability of fabrics should be assessed on a case-by-case basis. In some cases, conventional textiles may be more sustainable than smart textiles. For example, a cotton t-shirt made with organic cotton and dyed with natural dyes may be more sustainable than a smart textile t-shirt made with synthetic fibres and electronic components. Therefore, it is crucial to consider the entire life cycle of a textile and evaluate its sustainability based on its specific characteristics and intended use [15,16,17].

Green nanomaterials [13] can be integrated into smart textiles in various ways, depending on the intended application and the properties of the nanomaterials. Some standard methods for integrating green nanomaterials into smart textiles include coating (i.e., dip-coating, spray-coating, or electrospinning), embedding, printing (i.e., inject printing, screen printing, or gravure printing), or weaving [1,18].

Integrating nanomaterials into smart textiles increases concerns regarding their environmental impact and safety. Some environmental concerns include the potential for releasing nanoparticles during the production, use, and disposal of textiles, which can accumulate in the environment and affect ecosystems. Additionally, specific nanomaterials, such as silver nanoparticles, may have antimicrobial properties that could harm beneficial microorganisms in soil and water. The safety concerns are related to human exposure to nanoparticles through inhalation, ingestion, or skin contact, which may have toxic effects on human health. Furthermore, the long-term effects of exposure to nanoparticles still need to be fully understood, and more research is needed to determine their safety [18,19].

Green nanomaterials can address the environmental and safety concerns related to nanomaterials integrated into smart textiles in several ways. For example, biodegradable green nanomaterials can address concerns related to the persistence of nanomaterials in the environment. On the other hand, nanomaterials sourced from renewable sources such as plant extracts can reduce the use of non-renewable resources and reduce environmental impact. The safety concerns can also be addressed by non-toxic green nanomaterials that do not pose a risk to human health. Moreover, using sustainable synthesis methods such as green chemistry can contribute to reducing the environmental impact of nanomaterials. Thus, using green nanomaterials can reduce the environmental and safety concerns associated with nanomaterials integrated into smart textiles, making them a more sustainable and environmentally friendly choice [20,21].

Incorporating green nanomaterials can promote the sustainability of smart materials. As discussed earlier, green nanomaterials are produced using environmentally friendly methods and materials, and can be derived from renewable resources [21,22]. Smart textiles could also benefit from enhanced functionality from green nanomaterials, as such materials exhibit unique physical, chemical, and biological properties [18]. Aside from enhanced functionality in sensing, actuation, and energy harvesting, green nanomaterials can improve fabrics’ strength, flexibility, and durability. This can result in more comfortable, durable textiles, and resistance to wear and tear [22]. Using green nanomaterials in smart textiles can also promote health and safety. Traditional nanomaterials have raised concerns about their potential impact on human health and the environment. Green nanomaterials can reduce these concerns and ensure safe and responsible development of smart textiles. Moreover, incorporating green nanomaterials into smart textiles can also increase the market demand for these innovative and sustainable products. As consumers become more environmentally conscious and demand more sustainable products, smart textiles incorporating green nanomaterials can provide a competitive advantage to companies that produce them [23,24,25].

The purpose of a review regarding green nanomaterials for smart textiles dedicated to environmental and biomedical applications is to provide an overview of the recent advancements in the field of smart textiles and the potential use of green nanomaterials in their development. The review will focus on the synthesis, properties, and potential applications of green nanomaterials in smart textiles for environmental and biomedical applications, such as pollution monitoring, air and water filtration, drug delivery, wound healing, and disease diagnosis.

**Figure 1:**
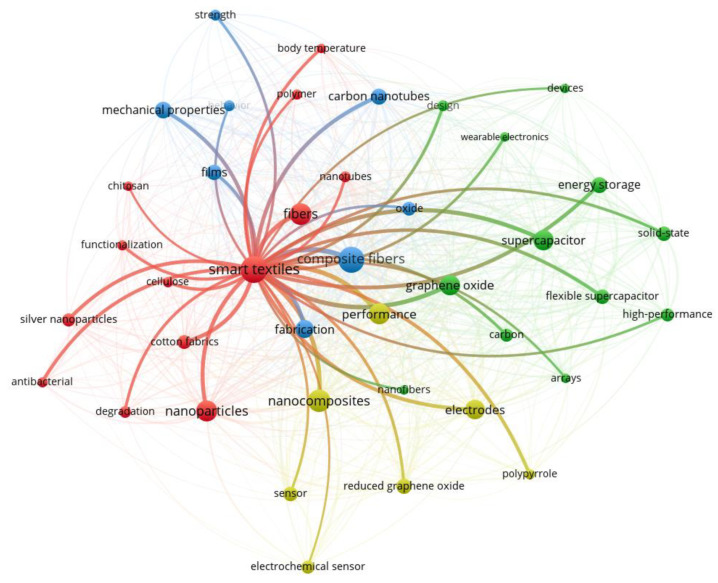
The bibliometric analysis of data extracted from the ISI Web of Science database using the keyword, “Green nanomaterials for smart textiles to environmental and biomedical applications”.

The analysis of the smart materials field (Figure 1) has been performed using a tool to visualise bibliometric elements, namely VOSviewer 1.6.18 [26]. From Figure 1, it can be seen that this area of interest is a very complex and interdisciplinary one.

VOSviewer is a powerful tool for analysing and visualizing networks based on keyword co-occurrence in the smart materials field. By interpreting the network analysis and visualization, we gain a deeper understanding of the relationships and trends within this rapidly evolving field. We use VOSviewer to identify clusters of related keywords within the network; we find clusters related to specific types of smart materials, such as shape memory alloys, supercapacitors, or nanocellulose.

The relevance of the items about smart materials presented in this review is associated with the number of times they appear in the ISI Web of Science database.

**Figure 2:**
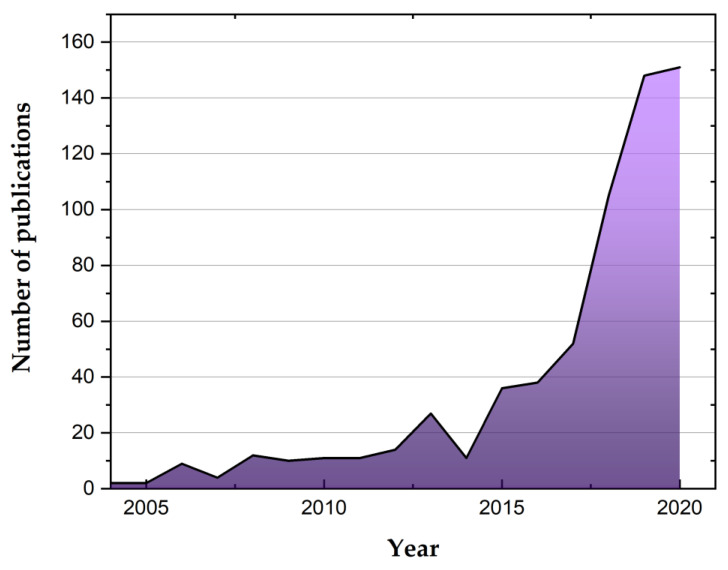
Publication trend (2000–2020) in the application of green nanomaterials for smart textiles (Source of raw data: ISI Web of Science; search keywords: “Green nanomaterials for smart textiles to environmental and biomedical applications”).

Figure 2 shows a bibliometric analysis of the data extracted from the ISI Web of Science (www.webofscience.com, accessed on 1 April 2023) database, using the following keywords: “Green nanomaterials for smart textiles to environmental and biomedical applications”.

The number of papers on the topic has increased considerably over the past five years, demonstrating the scientific community’s increased interest in the field.

In summary, incorporating green nanomaterials into smart textiles has many purposes and benefits, including promoting sustainability, enhancing functionality and performance, promoting health and safety, and meeting the growing market demand for sustainable and innovative textile products. The review will also address the challenges and limitations in developing and commercialising these materials, and future perspectives and opportunities in the field.

## 2. The Development of Sustainable and Environmentally Friendly Smart Textiles—An Overview

This review aims to provide insights into the development of sustainable and environmentally friendly smart textiles that can improve human health and the environment.

The limitations of this review regarding green nanomaterials for smart textiles dedicated to environmental and biomedical applications are related to the insufficient number of studies on the topic, as this is a relatively new and emerging field of research. Additionally, there are challenges in scaling up the production of green nanomaterials and integrating them into textile products. As the research on this topic is relatively new, the long-term performance, stability, and safety of green nanomaterials in different environmental and biomedical applications must be investigated.

Several reviews tackle this subject [1,18]. Some specific examples of green nanomaterials that can be integrated into smart textiles include cellulose nanocrystals [27,28], silver nanoparticles [29,30], alginate nanoparticles [30], chitosan nanoparticles or fibres [31,32,33], silk nanofibres, pullulan [34], clay nanoparticles (i.e., montmorillonite, kaolinite, and halloysite) [35,36], and the list may continue [37]. Table 1 illustrates the use of green nanomaterials integrated into smart textiles and their integration method.

## 3. Green Synthesis Methods for Nanomaterials Used for Smart Textiles

**Figure 3:**
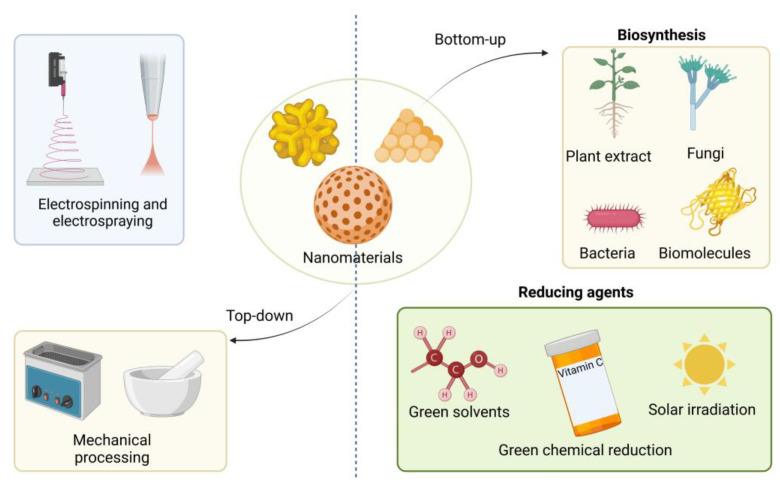
The top-down and bottom-up green synthesis methods of nanomaterials. (the graphical illustration has been created with BioRender software (https://www.biorender.com/about)—BioRender Company, Toronto, ON, Canada).

Green nanomaterial synthesis methods use eco-friendly and sustainable approaches, such as renewable resources, non-toxic solvents, and mild reaction conditions, and are popular due to their sustainability and eco-friendliness. These methods can also result in nanomaterials with unique properties and enhanced biocompatibility, making them attractive for biomedical applications. However, it is essential to ensure that the green synthesis methods are safe and effective, and that the resulting nanomaterials are thoroughly characterized and tested before use in any application [47,48,49,50,51]. The green synthesis of nanomaterials is addressed by bottom-up approaches (nanomaterials are assembled from individual atoms, molecules, or nanoparticles to form larger structures) in which bacteria, fungi, algae, and plant extracts are employed [52]. Top-down approaches are also used to synthesise green nanomaterials [53]. Figure 3 summarises the synthesis approaches of green nanomaterials, and each method is discussed in the following.

Biosynthesis involves using biological systems such as plant extracts (i.e., neem, green tea, aloe vera) [54,55,56], microorganisms (bacteria and fungi) [57,58,59], and biomolecules (proteins, enzymes, and carbohydrates) [60,61,62] as reducing or stabilizing agents to synthesize nanoparticles. Biosynthesis is a green and sustainable method, as it often requires mild conditions, low energy inputs, and nontoxic reagents [63,64].

Green solvents are environmentally friendly alternatives to the traditional solvents commonly used in the green synthesis of nanomaterials. They have several properties that make them attractive for green synthesis, including low toxicity, low volatility, high boiling points, and low environmental impact. Water, ethanol, and glycerol are polar solvents employed in the green synthesis of nanomaterials. Ionic liquids are salts in a liquid state at room temperature and have low volatility, high thermal stability, and tunable solubility. Supercritical fluids are gases compressed to a critical point, resulting in a substance with properties of both a gas and a liquid [65,66,67,68].

Green chemical reduction employs environmentally friendly reducing agents and solvents to synthesize nanomaterials. For instance, graphene oxide can be reduced to graphene using green reducing agents such as ascorbic acid instead of hazardous chemicals such as hydrazine [69]. This method minimises the use of toxic chemicals and reduces waste generation. Huang et al. [70] discussed graphene-based composites’ synthesis, properties, and applications, including a section on green reduction methods for graphene oxide.

Solar irradiation can be used in nanoparticle synthesis, as it acts as a reducing agent and energy source. This method is typically carried out under mild reaction conditions and requires no toxic chemicals [71,72].

Mechanical methods, such as high-energy ball milling or ultrasonication, can be used to produce green nanomaterials without the need for harsh chemicals or high temperatures. Cellulose nanofibres can, for example, be obtained from plant sources by mechanical processing, which involves grinding or refining the cellulose fibres for their separation and individualisation into nanofibres. Zhu et al. [73] presented various methods for the grinding and refining of cellulose nanofibres from wood. Abitbol and co-workers [74] provided an overview of nanocellulose materials, their properties, and their applications, including a discussion of mechanical processing methods for producing cellulose nanofibres. Chen et al. [75] investigated using ultrasonication combined with a mild chemical treatment to isolate cellulose nanofibres from various plant sources.

Electrospinning and electrospraying are other top-down methods that use electric fields to produce nanofibres or nanoparticles from solutions or melts of polymers, biopolymers, or other materials. Using green materials, such as chitosan or cellulose, and environmentally friendly solvents, electrospinning and electrospraying can create green nanomaterials for smart textiles. Bhardwaj et al. [76] provided an overview of electrospinning techniques for producing fibres from biopolymers such as chitosan and cellulose. Geng and co-workers [77] investigated the electrospinning of chitosan nanofibres using concentrated acetic acid as a green and environmentally friendly solvent.

Template-assisted synthesis involves using a porous membrane or a self-assembled monolayer as a template to guide the formation of nanomaterials with specific shapes and sizes. By using biodegradable polymers or naturally occurring structures as green templates, together with environmentally friendly synthesis conditions, the technique can create green nanomaterials for smart textiles [78,79].

The sol–gel process is another technique which can be adapted for the green synthesis of nanomaterials. The technique involves the formation of a colloidal suspension (sol) and the subsequent gelation of the sol to form a network structure (gel). By using biopolymers or metal-organic compounds as green precursors, and environmentally friendly solvents, the sol–gel process can be used to create green nanomaterials for smart textiles, such as stimuli-responsive hydrogels or biodegradable porous materials [80,81].

Green nanocomposites can be created by in situ polymerization, melt blending, or solution casting by combining green nanomaterials with biopolymers, natural fibres, or other environmentally friendly materials. Green nanocomposites can be used to create smart textiles exhibiting biodegradability, antimicrobial activity, or mechanical strength [82,83].

The methods exemplified above for obtaining green nanomaterials can promote the development of sustainable and eco-friendly smart textiles.

The way nanomaterials and textile substrates interact depends on their nature. When using green nanomaterials, physical adsorption can occur through van der Waals forces, electrostatic interactions, or hydrogen bonding. Metallic nanoparticles, for instance, can be physically anchored to the textile surface through sonochemical processes [84,85]. PEDOT:PSS, on the other hand, physically adheres to the textile fibres through a combination of mechanisms, including electrostatic forces and hydrogen bonding. The negatively charged sulfonate groups (SO_3_^−^) present in the PSS component of PEDOT:PSS can form electrostatic interactions with the positively charged sites on the textile fibres [86,87]. In another study, Jain et al. discovered that the entropy gain drives adsorption onto cellulose surfaces [88]. These interactions are based on weak forces that are reversible. Alternatively, nanomaterials and textiles can form covalent bonds, wherein electron pairs are shared between the atoms of both materials, resulting in a strong and stable attachment. Covalent bonding ensures that the nanomaterials remain securely anchored to the textile even when exposed to washing, mechanical stress, or chemicals [89]. For instance, Korica et al. conducted a study [90] wherein they treated viscose fabrics with 2,2,6,6-tetramethylpiperidine-1-oxy radical (TEMPO) and coated them with TEMPO-oxidized cellulose nanofibrils (TOCN). This process facilitated the covalent bonding of chitosan nanoparticles to the textile fibre by incorporating functional groups such as COOH and CHO. In their study, Xu and colleagues [91] utilised a pad-dry-cure technique to create cotton fabrics with antibacterial and ultraviolet (UV) protection properties. This was achieved through the use of carboxymethyl chitosan (CMCh) and Ag/TiO_2_ composites. By applying heat (at 180 °C) during the deposition process, the carboxyl group of the CMCh chain reacted with the hydroxyl group of the cotton cellulose to establish esterification. As a result, CMCh was covalently grafted onto the cotton fabric. The choice of interaction mechanism depends on the smart textile’s specific application and desired properties. Sometimes, a weaker or reversible interaction may be preferred to allow for easy modification or reusability. The most stable interaction mechanism is selected based on the compatibility between the nanomaterials and the textile substrate, as well as the surface chemistry and morphology of both materials. Table 2 reflects more examples of the textile substrates, the interaction mechanisms between textiles and nanomaterials, and the integration method.

## 4. Green Nanomaterials with Potential Applicability for Smart Textiles

Natural (i.e., cotton, silk, wool, and jute) and artificial fibres (i.e., polyester, nylon, and elastane) can be equally employed in smart textile development [99]. They provide the necessary structure, comfort, and functionality, which can be further enhanced by incorporating nanomaterials, electronic components, or sensors. Conventional materials used for smart textile development include conductive polymers (i.e., polyaniline—PANI or poly(3,4-ethylenedioxythiophene)—PEDOT), widely used to create flexible and stretchable conductive paths for electronic components and sensors. Metal wires and threads (i.e., copper or silver) are used to create electrical connections in smart textiles. They can be woven or embroidered into the fabric to enable conductivity and the transmission of electrical signals [100].

**Figure 4:**
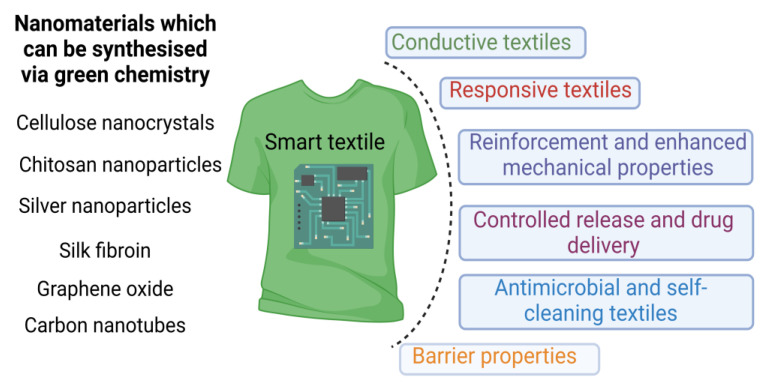
Green nanomaterials which can potentially be integrated into textiles and applications of smart textiles. (the graphical illustration has been created with BioRender software (https://www.biorender.com/about)—BioRender Company, Toronto, ON, Canada).

Conventional materials for smart textiles are often derived from fossil fuels and produced using energy-intensive processes. They may also involve toxic chemicals, leading to higher environmental pollution. Additionally, they do not possess the same level of biocompatibility as green nanomaterials, having the potential to cause allergic reactions or adverse effects on the skin, limiting their use in certain biomedical applications. In this context, green nanomaterials offer a promising solution for developing sustainable and environmentally friendly smart textiles. Recently, the potential applicability of green nanomaterials in smart textiles has been explored, including cellulose nanocrystals, chitosan nanoparticles, and silver nanoparticles synthesized from plant extracts. Figure 4 summarises some of the nanomaterials which can be synthesised via green chemistry and their potential applications in smart textiles.

Cellulose nanocrystals (CNCs) are biodegradable nanomaterials derived from cellulose and can be produced from wood, cotton, and bacteria. CNCs possess high strength, low density, biodegradability, and biocompatibility, making them attractive materials for smart textiles [101,102]. Green synthesis of CNCs involves the use of environmentally friendly methods to produce cellulose nanocrystals from plant-based materials [103,104]. The green synthesis of CNCs involves cellulose extraction, accomplished by acid hydrolysis, alkali treatment, and enzymatic hydrolysis [105]. Once the cellulose is extracted, it is processed to isolate the CNCs using mechanical processes such as high-pressure homogenisation or sonication, or by chemical processes such as acid hydrolysis [106]. The final step involves the characterisation of the cellulose nanocrystals to determine their size, morphology, and surface charge [105]. Bacteria, fungi, and algae have also been used to produce cellulose nanocrystals [107].

The process for obtaining CNCs from microorganisms is similar to that of plant-based materials. The microorganisms are grown under controlled conditions to produce cellulose, which is then extracted and processed to obtain the cellulose nanocrystals. One advantage of using microorganisms is that they can be grown in a controlled environment, producing uniform and high-quality CNCs [108]. Additionally, bacteria can produce cellulose more rapidly than plants, making them a potentially more efficient source of CNCs [109].

Some potential applications of CNCs in smart textiles are discussed in detail in the following. CNCs can be incorporated into textile fibres or fabrics to improve their mechanical properties, such as tensile strength, toughness, and elasticity. This can be beneficial in creating high-performance textiles for use in sports, military, and aerospace applications [110,111,112]. These nanomaterials could change their structure in response to temperature, humidity, or pH. Incorporating CNCs into textiles makes it possible to create fabrics that change their properties or appearance in response to external stimuli, allowing for the developing of smart textiles with tuneable properties [113,114]. Electrically conductive textiles can be created by combining CNCs with conductive materials (i.e. carbon nanotubes, graphene, or conductive polymers). These conductive textiles can create wearable sensors, electronic components, or energy-harvesting devices [115]. CNCs can form thin films or coatings on textiles to enhance their barrier properties against water, gases, or other environmental factors. This can be useful for creating protective clothing or packaging materials [116,117]. CNCs can be functionalized with antimicrobial agents or photocatalytic materials to create textiles with antimicrobial or self-cleaning properties. These textiles can help prevent the growth of bacteria and fungi, or break down organic stains and pollutants on the fabric’s surface, making them suitable for healthcare, sportswear, and military applications [118]. These green nanomaterials can be employed as a carrier for drugs or other therapeutic agents and integrated into smart textiles. These textiles can be used as wound dressings or wearable drug delivery systems, providing controlled and targeted release of the loaded agents [119,120]. As the demand for sustainable and environmentally friendly materials increases, the use of cellulose nanocrystals in smart textiles is likely to continue to grow.

Chitosan nanoparticles can be used as a coating to provide antimicrobial properties, or as an additive to improve the mechanical properties of the textile. Chitosan [121,122] is a biopolymer derived from chitin, found in crustaceans’ or shrimps’ exoskeletons [123,124]. Chitosan nanoparticles have been explored for their potential use in smart textiles thanks to their unique biocompatibility, biodegradability, and antimicrobial activity [125]. Green synthesis of chitosan nanoparticles involves using environmentally friendly methods to produce nanoparticles from chitosan [126,127,128]. The basic steps for the green synthesis of chitosan nanoparticles are the extraction of chitosan from chitin using an environmentally friendly method such as acid-free deacetylation or enzymatic hydrolysis. Chitosan is dissolved in an appropriate solvent, such as acetic acid, to form a chitosan solution, which is then added dropwise to a non-solvent (i.e., sodium hydroxide or sodium sulphate), under stirring conditions to form a nanoparticle suspension. The final step involves the characterization of the chitosan nanoparticles to determine their size, morphology, and surface charge [126].

Using environmentally friendly methods reduces the environmental impact of the synthesis process. Chitosan nanoparticles are biocompatible, making them suitable for delivery and tissue engineering applications [129]. The potential applications of chitosan nanoparticles are further discussed below.

Chitosan nanoparticles can be woven into textile fibres or used as a coating on the surfaces of fabrics to impart antimicrobial qualities, making these materials appropriate for use in healthcare, athletic apparel, and military settings [129,130,131,132]. They can be incorporated into intelligent fabrics and loaded with medications or other medicinal substances. Wound dressings and wearable drug delivery devices are potential applications for these materials [33,133,134]. Chitosan nanoparticles can be mixed with other conductive elements, such as carbon nanotubes, silver nanoparticles, or conductive polymers, to make conductive textiles. These fabrics have the potential to be fabricated into wearable sensors, heating elements, and other electrical components [135,136]. Chitosan nanoparticles can be loaded with colour-changing dyes or pigments, which can subsequently be incorporated into textiles. Chitosan nanoparticles can also be loaded with other types of pigments. These textiles can change colour in response to environmental conditions such as temperature, humidity, or exposure to UV light [137,138]. As a result, they can provide either visual feedback or concealment capabilities. Chitosan nanoparticles can encapsulate a wide variety of active substances, including phase change materials (PCMs) and scents. After being encapsulated, these particles can subsequently be included into textiles in order to provide temperature regulation, odour control, or other functionalities [139,140]. The use of chitosan nanoparticles in smart textiles is expected to grow as the demand for sustainable and environmentally friendly materials increases.

Silver nanoparticles (AgNPs) are a widely researched nanomaterial due to their excellent electrical conductivity, antimicrobial activity, and optical properties. Silver nanoparticles can be used as a coating to provide antimicrobial properties or as an additive to enhance the electrical conductivity of the textile [141]. Green synthesis of silver nanoparticles involves using eco-friendly methods to produce nanoparticles from silver ions. This method is becoming increasingly popular as it is more sustainable and has a lower environmental impact than traditional methods [142,143,144,145]. Plant-extract-mediated synthesis, microbial synthesis, biopolymer-mediated synthesis, and green-chemistry-mediated synthesis are several methods for obtaining AgNPs. Plant-extract-mediated synthesis involves using plant extracts (i.e., *Aloe vera*, green tea, and neem) as reducing and stabilizing agents to synthesize AgNPs [146,147,148]. Microbial synthesis involves the use of bacteria and fungi to synthesize AgNPs. The microorganisms produce enzymes and metabolites that can reduce and stabilize silver ions to form AgNPs [48,149]. Biopolymer-mediated synthesis uses chitosan and starch as reducing and stabilising agents to synthesize AgNPs [150]. Green-chemistry-based synthesis involves water as a solvent and glucose as a reducing agent [151]. The potential applications of green-synthesised AgNPs are summarised further.

Because AgNPs have antimicrobial properties, they can be incorporated into textile fibres and fabrics or applied as a coating on the surfaces of fabric. Because of this, they are suitable for use in healthcare settings, sportswear, and military uniforms to prevent infections and odours [152,153,154,155,156]. Because AgNPs have such high electrical conductivity, they can be used to make electrically conductive textiles [157,158]. These textiles can then be utilised to produce wearable sensors, touch-sensitive fabrics, flexible electronics, and heating elements. The fabrication of strain sensors, pressure sensors, or biosensors in smart textiles can be accomplished using silver nanoparticles. These sensors can be utilised for a variety of monitoring purposes, including detecting health metrics, monitoring environmental conditions, and monitoring body movements [159,160]. When mixed with phase change materials (PCMs) or other thermoregulating substances, silver nanoparticles (AgNPs) can be used to assist in the production of textiles that can regulate temperature. Because AgNPs have a high thermal conductivity, they are able to efficiently distribute heat throughout the fabric, which results in more accurate temperature regulation [161,162]. Materials that change colour can be made with the help of silver nanoparticles thanks to the plasmonic capabilities of these tiny particles. These fabrics can alter their colour in response to various stimuli, such as being exposed to different types of light or experiencing shifts in their immediate environment [18,163,164,165]. The usage of AgNPs allows for the production of textiles that provide increased protection against electromagnetic interference (EMI) as well as ultraviolet radiation. These materials have the potential to give a higher level of protection in outerwear and cases for electronic devices [166]. Notably, the use of AgNPs in textiles should be carefully considered due to their potential environmental and health impacts, even though they are synthesized via the green route.

Carbon nanotubes (CNTs) are a unique class of materials with a wide range of potential applications in areas such as electronics, energy storage, and biomedical engineering. One of the challenges in producing CNTs is finding environmentally friendly and cost-effective synthesis methods [167]. The following paragraphs present some examples of green synthesis methods for CNTs, and their potential applications in the textile industry. Qasim et al. [167] reviewed the use of plant-based materials as carbon sources for CNT synthesis. For example, it has been reported that spices can be used to synthesise CNTs with good optical properties [168]. Some bacteria can synthesise CNTs as a by-product of their metabolic processes. This method is auspicious because it is low-cost and environmentally friendly [169]. Hydrothermal synthesis involves using water at high temperatures and pressures to synthesise CNTs. This method is environmentally friendly because it does not require toxic chemicals or solvents [170].

Carbon nanotubes can be embedded within the fibres of the textile to provide conductivity or energy harvesting capabilities [171,172]. Research on using carbon nanotubes in smart textiles is ongoing, and new applications and technologies are continually being developed. For example, CNTs can be utilised to reinforce and enhance the mechanical properties for polymer composites, in structural health monitoring, in electromagnetic interference shielding, or in lightning strike protection [173].

Graphene oxide (GO) is a material with high mechanical strength, as well as thermal and electrical conductivity. It can also be used for drug delivery, as it can be functionalized with therapeutic agents [174,175,176]. Green synthesis methods for GO involve using natural sources and environmentally friendly materials for the synthesis process to minimise the environmental impact of GO production. The Hummers’ method is commonly used for synthesizing GO but involves employing strong acids and oxidizing agents that can harm the environment. Researchers have modified the Hummers’ method to make it more environmentally friendly by using hydrogen peroxide or potassium permanganate as alternative oxidising agents. This modification significantly reduces the amount of hazardous waste generated during synthesis [177]. The use of graphene oxide recently gained interest in the case of developing electronic textiles for biomedical applications [178].

Due to their antimicrobial properties, zinc oxide nanoparticles have been used for wound healing applications. They can also be used for drug delivery, as they are biocompatible and biodegradable [179,180]. Plant-extract-mediated synthesis uses extracts as reducing and capping agents to synthesise ZnO nanoparticles. *Aloe vera*, palm pollen, dried leaves, and other zinc hyperaccumulator plants can be used to synthesise ZnO nanoparticles [181].

Silk fibroin is a biocompatible and biodegradable protein derived from silk [182]. Silk fibroin nanoparticles have been used for drug delivery, as they can be functionalised with therapeutic agents [183,184].

This chapter emphasised the different green nanomaterials developed for biomedical and environmental purposes, which can be included in textile fabrics. These materials’ versatility and unique properties make them attractive for various applications, from wound healing to drug delivery to diagnostic tools.

## 5. Environmental and Medical Applications of Smart Textiles with Green Nanomaterials

**Figure 5:**
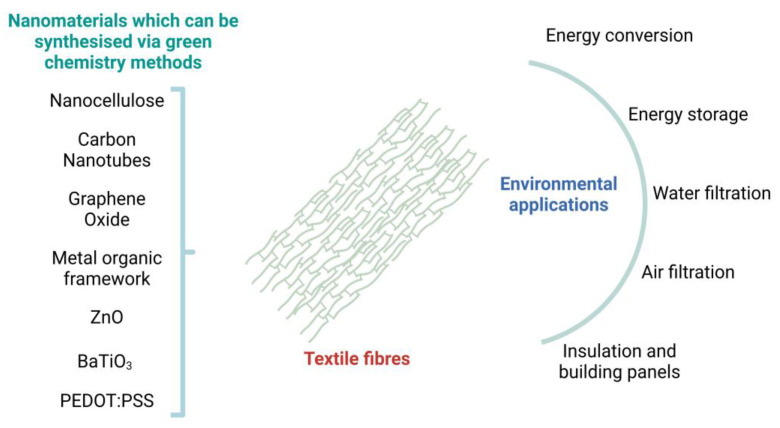
Nanomaterials which can be synthesised via green chemistry and potential environmental applications of the textile fibres incorporating green nanomaterials. (the graphical illustration has been created with BioRender software (https://www.biorender.com/about)—BioRender Company, Toronto, ON, Canada).

Smart textiles incorporating green nanomaterials can have various environmental applications, including air and water filtration, energy conversion and storage, and sustainable building materials [46,185,186]. These applications can potentially provide sustainable, cost-effective, and innovative solutions to environmental challenges (Figure 5).

Smart textiles incorporating green nanomaterials can be used for air filtration. For example, nanocellulose fibres have high filtration efficiency and low pressure drop, making them a practical and energy-efficient option for air filtration [187,188,189,190,191]. Jhinjer et al. [192] developed an in situ growth of zeolitic imidazolate metal-organic framework (ZIF MOF) on carboxymethylated cotton fabric for the adsorption of organic pollutants (aniline, benzene, and styrene). As Marino et al. presented [193], MOFs can be green-synthesised using *N*,*N*-dimethyl-9-decenamide, a bioderived solvent, as an alternative for structurally diverse MOFs.

GO and CNTs have been used to develop filters to remove heavy metals and bacteria from water. These filters can potentially provide a low-cost and sustainable option for water purification [194,195,196,197]. Xie et al. [198] studied a graphene oxide/Fe III based metal-organic framework membrane for water purification. Their developed membrane showed a photo-Fenton catalytic degradation efficiency of 98.81% for methylene blue (MB), and an efficiency of 97.27% for bisphenol-A (BPA). For water purification, carboxylated multi-walled carbon nanotubes (MWCNTs-COOH) loaded on cotton fabric acted as a separator of the evaporation layer from the bulk water [199]. Wang et al. developed an effective technique for textile wastewater purification based on MXene/Carbon nanotubes/Cotton fabric [200].

The textiles made with piezoelectric nanomaterials can convert mechanical energy, such as the movement of the fabric, into electrical energy. This can be used to power small electronic devices, such as sensors and wearable technology [192,194,201,202]. GO, ZnO, and BaTiO_3_ obtained by green chemistry synthesis methods can be incorporated into fabrics to generate electricity from the mechanical stress produced by body movements [203,204,205,206].

Textiles made with supercapacitive nanomaterials can store and release electrical energy quickly, making them a potential option for energy storage in wearable technology [1,18,194,207]. In this sense, Dou et al. [208] developed a strain sensor based on a weft knitted fabric with carbon nanotubes and polypyrrole deposited on the surface (WSP-CNT-PPy), with robust electrochemical and electro-heating properties. The strain sensor can be considered in future applications for wearable electronics. Poly(3,4-ethyeledioxythiopene) (PEDOT) doped with poly(styrenesulfonate) (PSS) can be employed as flexible textile supercapacitors. For example, Li et al. [209] used a spray-coating approach to create graphene nanosheets with PEDOT:PSS. The conductive fabrics demonstrated an increased specific surface capacitance of 245.5 mF/cm^2^, allowing them to be employed as flexible textile supercapacitors.

To enhance the effectiveness of photovoltaic devices in collecting photo-generated electrons, nanocomposites with high electron mobility can be designed rationally. Although single-walled carbon nanotubes possess exceptional electron mobility, it remains difficult to incorporate them into nanocomposites for efficient photovoltaic devices. In the study conducted by Dang et al. [210], the synthesis of nanocomposites consisting of single-walled carbon nanotube-TiO_2_ nanocrystal core-shell structures, utilizing a genetically engineered M13 virus as a template, was presented.

Smart textiles incorporating green nanomaterials can also be used in sustainable building materials. For example, textiles made with nanocellulose fibres can be used as insulation and building panels, reducing the need for traditional construction materials, and promoting sustainability [37,202,211].

Incorporating green nanomaterials into environmental applications such as air and water filtration, energy conversion and storage, and sustainable building materials can improve efficiency, reduce waste, increase sustainability, save costs, and improve performance. These advantages highlight the potential of green nanomaterials to provide innovative and sustainable solutions to environmental challenges [187,194].

**Figure 6:**
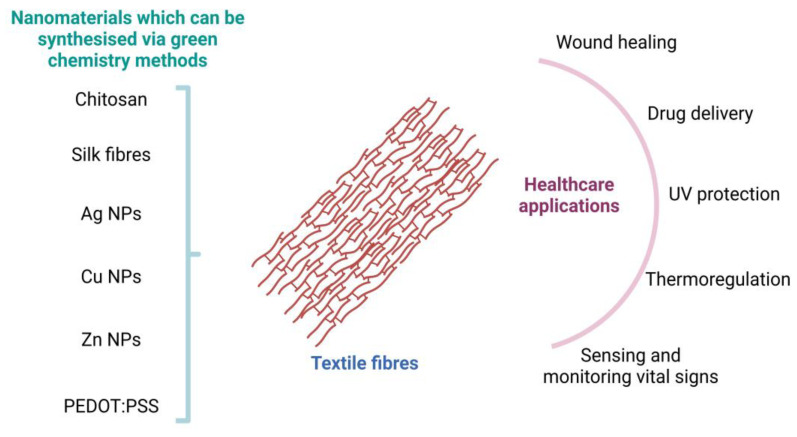
Nanomaterials which can be synthesised via green chemistry and potential healthcare applications of the textile fibres incorporating green nanomaterials. (the graphical illustration has been created with BioRender software (https://www.biorender.com/about)—BioRender Company, Toronto, ON, Canada).

Smart textiles with green nanomaterials have many potential health applications thanks to their unique properties and functionality (Figure 6).

One approach to creating smart textiles with green nanomaterials for wound healing is to coat fibres with antibacterial nanoparticles. For example, silk fibres coated with chitosan nanoparticles proved to have antibacterial properties and promote the growth of skin cells [182,212,213,214,215,216]. Chitosan nanoparticles have antibacterial properties against various bacteria, including MRSA (methicillin-resistant *Staphylococcus aureus*), *Escherichia coli*, and *Pseudomonas aeruginosa* [214,217]. A simple dipping method can be used to create silk fibres coated with chitosan nanoparticles. First, silk fibres are soaked in a solution containing chitosan nanoparticles. The fibres are then dried and heated to stabilise the coating. The resulting silk fibres are antibacterial and have been shown to promote the growth of skin cells, making them ideal for use in wound dressings [218]. The antibacterial properties of silk fibres coated with chitosan nanoparticles can help prevent wound infections while promoting skin cell growth and can aid in the healing process.

Additionally, silk fibres are biocompatible and biodegradable, which means they are safe for the body and will break down naturally over time [219]. Silver nanoparticles have also been shown to have strong antibacterial properties and are effective against a wide range of microorganisms, including antibiotic-resistant strains [220,221]. In summary, smart textiles with green nanomaterials, such as silk fibres coated with chitosan nanoparticles, have great potential for wound healing due to their antibacterial properties and ability to promote skin cell growth. Further research is needed to fully understand these materials’ potential benefits and risks in wound healing applications.

Smart textiles with green nanomaterials can be used for localised drug delivery [222]. Silver nanoparticles can be used to deliver antibiotics to the site of infection, reducing the number of antibiotics needed and minimising the risk of systemic side effects [220]. Cotton fibres can be coated with silver nanoparticles for drug delivery by electrospinning, dip-coating, and chemical vapour deposition. The antibacterial properties of the silver nanoparticles were also effective at killing bacteria in the surrounding area [38]. In a study [223], cotton fibres coated with silver nanoparticles have shown a good release of antibiotics over a sustained period, with the release rate varying depending on the thickness of the coating. Overall, smart textiles with green nanomaterials have the potential to revolutionize drug delivery by providing a targeted, localized approach to treatment. In the case of cotton fibres coated with silver nanoparticles, this technology can improve the effectiveness of antibiotic treatments while minimising side effects.

Smart textiles with green nanomaterials can be designed to provide UV protection for the skin. For example, cotton fibres coated with zinc oxide nanoparticles have been shown to provide UV protection while remaining breathable. UV protection is essential for textiles and clothing, as prolonged exposure to UV radiation can lead to skin damage and increase the risk of skin cancer [224,225]. Zinc oxide nanoparticles effectively absorb and scatter UV radiation, making them ideal for UV-protective textiles. The nanoparticles can be easily incorporated into textiles through various coating methods, such as dip-coating, spraying, or electrospinning [226]. Further research is needed to fully understand these materials’ potential benefits and risks in UV protection applications [225].

Temperature regulation is an essential consideration in many applications, particularly in healthcare. Smart textiles with green nanomaterials have potential applications in body temperature regulation, benefiting individuals with hypothermia or hyperthermia. For instance, copper nanoparticles embedded in fabrics can help regulate body temperature by reducing heat loss [18,227]. Copper nanoparticles have unique properties, including high thermal conductivity, which makes them effective at regulating body temperature. Copper nanoparticles can be embedded into fabrics using various methods, such as dip-coating or electrospinning [18].

Smart textiles with green nanomaterials can potentially be used for sensing and monitoring vital signs and biometric data in real time, helping in the early detection of health problems and preventing complications. For instance, Shamena et al. employed poly(3,4-ethyeledioxythiopene) (PEDOT) doped with poly(styrenesulfonate) (PSS) films, as they are used for electronic applications and are stable, biocompatible, and flexible. In addition, silver and copper nanoparticles were blended with PEDOT:PSS to provide better conductivity [228]. PEDOT:PSS can be synthesized via green synthesis methods. In situ polymerization of PEDOT:PSS containing differing tin oxide (SNO_2_) content in aqueous medium using plasma-activated H_2_O_2_ as oxidant has been reported [229]. In another scientific paper [230], negatively charged gold and silver nanoparticles, prepared using the dry leaf of *Annona reticulata*, were employed in PEDOT:PSS thin films. By electrospinning a mixture of poly(vinyl alcohol) (PVA) and PEDOT:PSS, Zhang et al. [231] created ultrafine conductive nanofibres with an average diameter of 68 nm. The resulting composite of PVA/PEDOT:PSS was employed as a sensor for detecting low concentrations of ammonia with high sensitivity.

For healthcare applications, incorporating green nanomaterials into smart textiles can provide numerous benefits, including antibacterial properties, which can help prevent the spread of infections in healthcare settings. Additionally, the incorporation of green nanomaterials has the potential to increase the performance and durability of the textile, resulting in longer-lasting, more comfortable textiles that can withstand repeated use and washing. Zinc oxide nanoparticles have been shown to have antimicrobial and anti-inflammatory properties, which can help improve wound healing. By incorporating these materials into smart textiles, wound dressings can be created that are more effective at preventing infections and promoting healing. Last but not least, green nanomaterials are typically non-toxic and biodegradable, making them safer for both the environment and human health.

## 6. Conclusions and Perspectives

Smart textiles incorporating green nanomaterials have the potential to add new functionalities to clothing and other textile products, such as sensing, actuation, and energy harvesting. Green nanomaterials, which are produced using environmentally friendly methods and materials, are particularly promising as they address the environmental concerns associated with traditional nanomaterials. Nanomaterials in smart textiles possess the potential to revolutionize topics, from environmental science to medicine, due to their unique properties. The future potential for the development and commercialization of these materials is expansive and diverse.

Scientists are investigating textiles incorporating carbon-based nanomaterials or metal-organic frameworks (MOFs), which could effectively filter pollutants, contaminants, and pathogens from air and water. The objective is to develop wearable technology that promotes personal and environmental health [232,233]. It is possible to create personal energy harvesting and storage systems through the development of textiles that incorporate nanomaterials such as photovoltaic cells, thermoelectric devices, and piezoelectric materials. This will increase the energy efficiency of wearable technology and lessen our reliance on conventional energy sources [234]. Real-time health monitoring is one of the most promising uses for smart textiles based on nanomaterials. Sensors can be incorporated into these textiles to monitor physiological variables, including heart rate, body temperature, and blood oxygen levels. Future breakthroughs might include the ability to identify diseases [235]. Future smart textiles may contain nanoparticles that may securely transport and deliver medications or other therapies, which could revolutionize the way we handle a range of medical issues [236,237]. Nanomaterials have the potential to significantly enhance wound treatment. To encourage more rapid recovery, decrease the risk of infection, and even encourage tissue regeneration, smart fabrics may be developed [238]. Despite these promising customers, there are still barriers to be solved for the use of nanoparticles in smart textiles in the future. While many lab-scale demonstrations have been successful, scaling these processes for mass production remains a challenge. The efficacy of the nanoparticles and their related features must be maintained throughout the life cycle of the textile product, including through washing and use. To ensure the reliability and effectiveness of these materials, appropriate standards and regulations must be developed, as with any breakthrough technology. It is important to fully understand and control how nanomaterials interact with the environment and the human body. In order for the public to easily understand and accept these products, education and communication are essential. Issues and scepticism about the usage of nanoparticles may exist, and these must be properly addressed.

Nevertheless, there are still several challenges to be addressed in developing and implementing smart textiles incorporating green nanomaterials, such as those related to compatibility, cost, safety, and regulation. However, the growing interest and investment in this field suggest that we can expect continued progress in developing these innovative and sustainable textile products in the future. Consumer acceptance, legal issues, cost-effectiveness, and the scalability of production of nanomaterials, as well as the capacity to integrate nanoparticles into textiles without affecting their qualities, will be crucial to commercialization. Sustainability is an additional important aspect of commercialization. From production to disposal, the life cycle of these materials should be evaluated and optimized to reduce their environmental impact. For instance, biodegradable or recyclable nanomaterials could be investigated.

## Figures and Tables

**Table 1:** The use of green nanomaterials integrated into smart textiles.

Textile	Nanomaterials	Synthesis Method	Integration Method	Application	Ref.
cotton	silver nanoparticles	green synthesis using seaweed extract (*Padina gymnospora)*	coating	Antibacterial and water-repellent textiles for healthcare and outdoor use	[38]
jute	silver nanoparticles	green synthesis using plant extract	ultraviolet (UV) photoreduction and by using polyethylene glycol as a reducing agent and stabilizer	Antibacterial and durable textiles for agricultural and industrial use	[39]
cotton	zinc oxide nanoparticles	green synthesis using plant extracts such as *Anisochilus carnosus and Plectranthus amboinicus*	sol–gel method with a green solvent	UV-resistant and antibacterial textiles for outdoor and healthcare use	[40]
cotton	copper oxide nanoparticles	green synthesis using green plant *Carica papaya* leaves	dispersion	A medical textile to avoid cross-infection within a clinical environment	[41]
antibacterial fabric	zinc oxide nanoparticles	green synthesis using *Moringa oleifera* extract	melt spinning, dry-jet wet spinning	Antibacterial and UV-protective textiles for healthcare and outdoor use	[42]
synthetic fibres	gold nanoparticles	green synthesis using *Lantana camara linn* leaf extract	dip coating, electroless, screen printing, dropwise, immersion, sonication, and electrospinning	Antimicrobial and conductive textiles for healthcare and wearable electronics	[43]
gelatine-bioactive glass	cellulose nanocrystals	green synthesis using *Komagataeibacter xylinus* bacterium	freeze-drying technique	Fabrication of synthetic bone tissue scaffolds with high compressive strength and wettability	[44]
poly(l-lactic acid)	chitosan nanoparticles	green synthesis using a natural biopolymer such as chitosan	casting	Antibacterial and durable textiles	[45]
cotton	TiO_2_ nanoparticles	green synthesis using *Azadirachta indica* leaf extract	immobilisation	Decontamination, self-cleaning of intense stains, and bacterial inhibition without TiO_2_ UV-activation	[46]

**Table 2:** Interaction mechanism between nanomaterials and textile substrates.

Textile Substrates	Nanomaterials	Interaction	Integration Method	Ref.
cotton	(CMCh) and Ag/TiO_2_ composites	Covalent, esterification between the hydroxyl group of cotton and carboxyl group of CMCh	pad-dry-cure	[91]
viscose	2,2,6,6-tetramethylpiperidine-1-oxy radical (TEMPO)-oxidized cellulose nanofibrils (TOCN)	Covalent, functional groups (COOH and CHO) suitable for irreversible binding of chitosan nanoparticles	TEMPO-mediated oxidation of native cellulose	[90]
cotton	AgNPs and PdNPs	Semi-covalent	impregnation with thiol-modified cellulose fabric	[92]
cotton	CeO_2_ nanoparticles	Non-covalent	immobilisation of CeO_2_ nanoparticles on a chitosan-treated linen fabric using in situ synthesis	[93]
non-Woven Fabrics	Nanocomposite Nylon 6/ZnO	Non-covalent	ultrasound-assisted Extrusion	[94]
wool-polyamide/polyester textiles	TiO_2_ nanoparticles	Non-covalent	wet chemical technique	[95]
polyester fabrics	Titania nanowires	Non-covalent	Sol–gel	[96]
cotton fabric	PANI/TiO_2_	Non-covalent	polymerization	[97]
cotton fabrics	Platinum (IV) chloride modified TiO_2_ and N-TiO_2_ nanosols	Non-covalent	dip-coating process	[98]

## Data Availability

Not applicable.

## Abbreviations

Au	Gold
Ag	Silver
WHO	World Health Organization
QD	quantum dots
ASTM	American Society for Testing and Materials
NP	nanoparticle
mm	millimetre
nm	nanometre
NM	nanomaterial
HPLC-MS	High-performance liquid chromatography-mass spectrometry
PCR	Polymerase chain reaction
ELISA	Enzyme-linked immunosorbent assay
Pt	Platinum
Pd	Palladium
Zn	Zinc
Cd	Cadmium
Cu	Copper
Fe	Iron
Ni	Nickel
Co	Cobalt
HAuCl_4_	Tetrachloroauric Acid
H_2_PtCl_6_	Hexachloroplatinic acid
RhCl_3_	Rhodium (III) chloride
PdCl_2_	Palladium (II) chloride
cm	centimetre
TiO_2_	*Titanium dioxide*
RF	radio frequency
K	Kelvin
kHz	kilohertz
MHz	megahertz
kW	kilowatt
MW	megawatt
atm	atmosphere
sec	seconds
N	Nitrogen
DMF	dimethylformamide
PEG	polyethylene glycol
UV	ultraviolet
AuNPs	gold nanoparticles
°C	degrees Celsius
min	minutes
ZnO	Zinc oxide
SnO_2_	Tin oxide
PbO	Lead (II) oxide
EC-SPR	Electrochemical—surface plasmon resonance sensor
DNA	Deoxyribonucleic Acid
LSPR	Localised surface plasmon resonance
SERS	Surface-enhanced Raman scattering
*E. coli*	*Escherichia coli*
PMNCs	polymeric nanocomposites
antibodies	ABs
GOX	glucose oxidase
PDA	polydopamine
DA	dopamine
CFU	colony-forming unit
mL	millilitre
PtNPs	platinum nanoparticles
PBNCs	polymeric bionanocomposites
*L. monocytogenes*	*Listeria monocytogenes*
μm	micrometre
LOD	Limit of detection
g	gram
β-Gal	β-galactosidase
*S. typhimurium*	*Salmonella typhimurium*
h	hours
PBS	phosphate buffered saline
EC	Commission Regulation
No	Number
*S. boydii*	*Shigella boydii*
ICS	immunochromatographic strip
*S. aureus*	*Staphylococcus aureus*
ATCC	American Type Culture Collection
MNPs	metal nanoparticles
MOs	metal oxides
CuO	copper oxide
Ag_2_O	silver oxide
CuNPs	Copper nanoparticles
pg	picograms
Fe_3_O_4_	Iron oxide
SeNP	Selenium nanoparticle
FeNP	Iron nanoparticle
kg	kilogram
K	Potassium
Mg	Magnesium
Ca	Calcium
Hg	Mercury
IC	inhibition concentration
LC	lethal concentration
CMT	maximum permissible concentration
FDA	Food and Drug Administration
LOx	lactate oxidase
BC	Bio-cellulose
Co	Collagen
CuONPs	Copper oxide nanoparticles
fmol	femtomole
COVID-19	Coronavirus Disease 2019
SARS-CoV-2	Severe acute respiratory syndrome coronavirus 2
USD	The United States dollar
LDPE	Low-density polyethylene
RFID	Frequencies radio
EFSA	The European Food Safety Authority
MNTS	Micro- and Nanotechnologies
LDPE	Low-density polyethylene
OR	oil of oregano
RO	rosemary oil
SWNT	single walled carbon nanotube based
PLL	Poly-L-lysine
ESI	electrospray ionisation
GCE	glassy carbon electrode
PCL	polycaprolactone
PHB	polyhydroxy butyrate
PHV	polyhydroxy valerate
PE	polymers polyethylene
PVC	polyvinyl chloride
EVOH	ethylene vinyl alcohol
IgG	Immunoglobulin G
IgM	Immunoglobulin M
PBAT	poly (butylene adipate-co-terephthalate
TPS	cellulose-based thermoplastic starch
PLA	poly lactide
PHA	poly-hydroxyalkanoate
PHB	poly-hydroxybutyrate
PGA	poly-glutamic acid
MCF-7	Michigan Cancer Foundation-7
MOF	metal-organic framework
ZIF MOF	zeolitic imidazolate metal-organic framework
MWCNTs-COOH	carboxylated multi-walled carbon nanotube
WSP-CNT-PPy	weft-knitted spacer fabric-carbon nanotubes-polypyrrole
SNO_2_	tin oxide
PEDOT	poly(3,4-ethylenedioxythiophene)
PEDOT:PSS	poly (3,4ethyeledioxythiopene) doped with poly(styrenesulfonate)
PVA	poly(vinyl alcohol)
TEMPO	treated viscose fabrics with 2,2,6,6-tetramethylpiperidine-1-oxy radical
TOCN	TEMPO-oxidized cellulose nanofibrils
CMCh	carboxymethyl chitosan
PANI	polyaniline
